# Hostile clinician behaviours in the nursing work environment and implications for patient care: a mixed-methods systematic review

**DOI:** 10.1186/1472-6955-12-25

**Published:** 2013-10-04

**Authors:** Marie Hutchinson, Debra Jackson

**Affiliations:** 1 School of Health and Human Sciences, Southern Cross University, PO Box 157, Lismore 2780, Australia; 2 Faculty of Health, University of Technology, PO Box 123, Broadway Sydney, Australia

**Keywords:** Workplace bullying, Disruptive behaviour, Quality of care, Teamwork, Nurse-physician relations, Work environment, Systematic review

## Abstract

**Background:**

Although there is a sizeable body of evidence regarding the nature of hostile behaviours among clinicians in the nursing workplace, what is less clear is the nature of the relationship between these behaviours and patient care. To inform the development of appropriate intervention strategies we examine the level of evidence detailing the relationships between hostile clinician behaviours and patient care.

**Methods:**

Published qualitative and quantitative studies that examined hostile clinician behaviours and patient care were included. Quality assessment, data extraction and analysis were undertaken on all included studies. The search strategy was undertaken in July and August 2011 and comprised eight electronic databases (CINAHL, Health Collection (Informit), Medline (Ovid), Ovid Nursing Full Text, Proquest Health and Medicine, PsycInfo, Pubmed and Cochrane library) as well as hand searching of reference lists.

**Results:**

The search strategy yielded 30 appropriate publications. Employing content analysis four themes were refined: *physician-nurse relations and patient care, nurse-nurse bullying, intimidation and patient care, reduced nurse performance related to exposure to hostile clinician behaviours, and nurses and physicians directly implicating patients in hostile clinician behaviours.*

**Conclusions:**

Our results document evidence of various forms of hostile clinician behaviours which implicate nursing care and patient care. By identifying the place of nurse-nurse hostility in undermining patient care, we focus attention upon the limitations of policy and intervention strategies that have to date largely focused upon the disruptive behaviour of physicians. We conclude that the paucity of robustly designed studies indicates the problem is a comparatively under researched area warranting further examination.

## Background

Internationally, various studies and reports have raised concern regarding hostile behaviours in the nursing workplace [1-3]. Although aggression and violence from patients and their visitors are cause for concern, nurses report hostility from colleagues, managers and other professionals to be of most concern [4]. The types of behaviours that constitute hostile behaviours between clinicians have been variously categorised as horizontal or lateral violence, insider perpetrated violence, relational aggression, bullying, incivility, harassment, and aggression [5-7]. The instigators of hostile clinician behaviours in the nursing context can include other nurses, physicians or other health professionals [8]. These various forms of behaviour less commonly include physical violence and are more likely to involve verbal abuse, continual criticism, demeaning remarks, intimidation, threat of harm, physical assault, sexual harassment and undermining, as well as more subtle behaviours such as refusing to cooperate, withholding clinical information, being unavailable to give assistance, hampering another’s performance and making their work difficult [7,9].

It is widely held that the various forms of hostile behaviour exhibited in the workplace threaten patient safety by impacting negatively upon the nature of the work environment, including eroding effective professional communication and professional relationships that underpin delivery of safe care [10-12]. Hostile behaviour within work teams can result in reduced communication and disruption to teamwork [8]. In the health care context, relatively subtle forms of hostile clinician behaviours, such as withholding information or covert intimidation have the potential to cause serious harm when patient care is impacted [13]. Furthermore, these behaviours not only have the potential to impact negatively upon the delivery of patient care, the consequences for clinicians targeted by hostility can include anxiety, depression, post-traumatic disorder and withdrawal from work [1].

Reflecting the degree of concern about hostile workplace behaviours, The Joint Commission responsible for accrediting health care organizations in the US released a Sentinel Event Alert drawing attention to the dangers of intimidation and disruptive behaviours in undermining a culture of safety [14]. The dissemination by the Joint Commission of a Sentinel Event Alert reflects concern about an identified trend in sentinel events related to unexpected patient death or serious injury. While the term ‘disruptive behaviour’ was initially used to describe behaviours between clinicians that had the capacity to undermine safety or disrupt care [14,15], ambiguity around the meaning of this term saw a move towards the umbrella term ‘behaviours that undermine a culture of safety’ [16]. Importantly, both of these terms minimise the hostile, and often aggressive, intimidating and abusive nature of the behaviours involved, which can include physical assault, bullying, sexual harassment and racial slurs [17]. For this reason in this manuscript we have chosen the less ambiguous term hostile clinician behaviours as an umbrella concept to encapsulate the range of hostile behaviours that may occur between clinicians and contribute to adverse patient care by influencing the culture of safety, quality of care and patient satisfaction [14].

Despite widespread recognition of the potential impact of hostile clinician behaviours on patient care, no studies have systematically examined the available evidence on the relationships between these factors.

### The review

#### Aims

For this review we were specifically interested in examining the relationship between the various forms of hostile clinician behaviours and patient care. The following two questions were developed to guide the systematic review and analysis. (i) Do hostile clinician behaviours negatively influence patient care? and (ii), If so, how do these behaviours impact aspects of care delivery?

The various forms of behaviour included under the umbrella concept of hostile behaviours include aggression and threat of harm, physical violence, verbal abuse, bullying, horizontal violence and lateral hostility, intimidation and harassment, as well as more subtle forms of uncivil or disruptive behaviours such as refusing to co-operate, withholding information, being unavailable to give assistance, hampering another’s performance and making their work difficult [7,9].

## Methods

Papers published during the period 1990–2011 were included in the review. This timeframe was chosen following an initial scoping review of the literature that identified the emergence of literature on hostile clinician behaviours in the nursing context from the early 1980s, with reported studies appearing after 1990. To provide more in-depth understanding into the questions the review sought to address a mixed-studies design, including qualitative, quantitative, and mixed methods studies was chosen [18]. The initial scoping review identified that there has been very little primary research on this topic, thus the scope of the review was enhanced by examining data from studies not primarily designed to investigate the relationship between hostile clinician behaviours and clinical outcomes. This type of review is useful where there is limited research on an important issue to inform policy and practice.

### Outcomes of interest

For the purpose of the review, patient care refers to care processes or events. It includes the influence of clinician behaviours and attitudes as well as interventions upon clinical care, safety and quality. Hostile clinician behaviour refers to various forms of rude, intimidating, hostile, aggressive, uncivil, harassing, bullying, or disruptive behaviours occurring between clinicians. In the nursing lexicon these behaviours when they occur between nurses are commonly labelled oppressed group behaviour and horizontal or lateral violence.

### Search strategy

The search strategy included eight electronic databases (CINAHL, Health Collection (Informit), Medline (Ovid), Ovid Nursing Full Text, Proquest Health and Medicine, PsycInfo, Pubmed and Cochrane library) and was undertaken in July and August 2011. Titles, abstracts and subject descriptors were searched using the keywords - *bullying, incivility, violence, horizontal violence, lateral violence, disruptive behaviour, aggression,* in combination with nurs*, physician, doctor, patient care, clinical care, adverse events, patient safety, and quality. Hand searching of reference lists was also undertaken.

### Inclusion criteria

**Figure 1:**
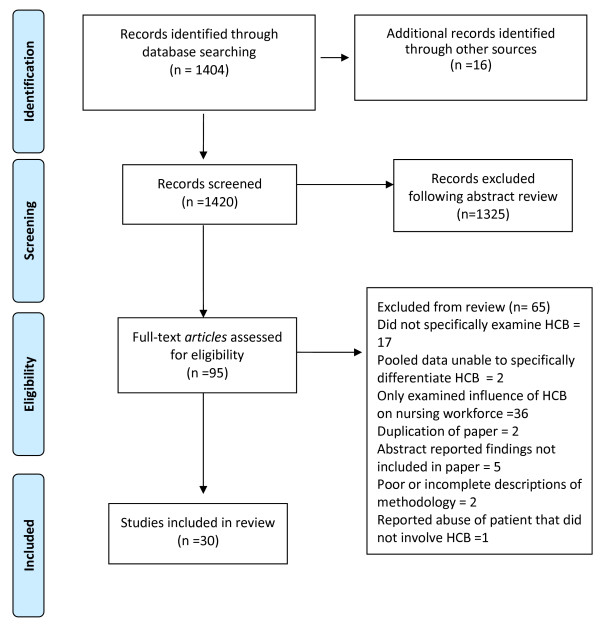
Summary of search process.

Following review, abstracts were selected if they met the following inclusion criteria: (1) peer reviewed research, (2) manuscripts published in English (3) unpublished masters or doctoral thesis, (4) substantive reviews, (5) studies that examined the relation between one or more forms of hostile clinician behaviour experienced by nurses and patient care, (6) studies that examined the relation between hostile clinician behaviours and features of the nursing work environment and patient care. Through the initial screening process 106 manuscripts were identified for scrutiny. Excluded from this pool were studies of faculty incivility and cross sectional survey studies that did not specifically identify the relations between hostile clinician behaviours and patient care. Following this initial process 95 manuscripts were retrieved for full reading. Of these 95 the majority of those excluded reported the impact of hostile clinician behaviours upon nurses or the nursing work environment without evidence of any association with patient care (n = 36) or did not specifically examine hostile clinician behaviours (n = 19), with two further studies of workplace violence excluded as pooled data in the samples meant it was not possible to isolate the effect of clinician behaviours from other sources of violence i.e. patients and visitors. Additional reasons for exclusion included: duplication of data presented in another paper included in the review (n = 2); abstract reported findings that were not included in the paper (n = 5); poor or incomplete descriptions of the methodology (n = 2); and, reported abuse of patients which did not involve hostile behaviour between clinicians (n = 1). The search strategy (summarised in Figure 1) eventually yielded 30 appropriate publications [7,8,15,19-42] comprising 19 survey studies, 2 mixed methods studies and 9 qualitative studies. Detail on the studies included in the review is summarised in Table 1.

**Table 1:** Studies included in the review

**Author(s) country**	**Setting or context**	**Aim(s) of the study**	**Design and sample**
Curtis, Bowen et al. [19] Australia	Students enrolled in one university nursing program	Student nurses experience of horizontal violence	Non-randomised cross sectional sample with open ended responses
			152 Student nurses
Corney [20] Australia	Registered nurses	Nurses experience of aggression	Qualitative research Heideggerian methodology.
			Sample 2 nurses
Chairella and McInnes [43] Australia	Nursing case law	Explore legal and ethical frameworks that inform nursing practice	Review of case law 1904–2002 pertaining to nursing
			180 cases reviewed
Farrell [21] Australia	Registered nurses	Nurses experience of aggression in the clinical setting	Sequential mixed method
			Non-randomised cross sectional survey. Sample 270
			Qualitative component -Grounded theory. Sample 29 nurses (n = 7 university lecturers, 20 clinical staff & 7 university lecturers)
Fasolino and Snyder, [22] United States	Nurses in 1 hospital	Relationships between nurse practice environment and medication errors	Non-randomised cross sectional survey
			Sample 248 RNs
Gunnarsdo’ttir et al., [23] Iceland	Nurses in one hospital	Aspects of nurses’ work environment linked with job outcomes and assessments of quality of care	Non-randomised cross sectional survey
			Sample 695 nurses
Hanrahan et al., [24] United States	Psychiatric registered nurses 67 general hospitals	Relationship between nurse practice environment and adverse events	Non-randomised cross sectional survey linked to secondary hospital data
			Sample 353
Tervo - Heikkinen et al., [25]	Registered nurses 34 inpatient wards	Relationship between nurse’s work environment and nursing outcomes	Non-randomised cross sectional survey
			Sample 664
Higgins and MacIntosh [26] Canada	OR nurses	Nurses perceptions of physician perpetrated abuse	Qualitative research
			Purposive sample from cohort of randomly selected nurses n = 10
Hutchinson, Vickers et al. [7] Australia	Registered and Enrolled nurses	Nature and extent of bullying in the Australian nursing workplace	Qualitative research
			Convenience sample n = 26 nurses
Institute for Safe Medication Practices [27] United States	Health care providers	Not specified	Non-randomised cross-sectional Sample N = 2,095
			(1,565 nurses, 354 pharmacists, 176 others)
Jackson, Peters et al. [28] Australia	Registered and Enrolled nurses	Experiences of nurse whistleblowers	Qualitative research Narrative Inquiry
			Non-randomised convenience sample n = 18
Lyndon, [29] United States	Registered nurses (RNs), physicians (MDs), and CNMs 2 hospitals	Interpersonal, structural, and social processes affecting individual and collective among nurses and physicians	Qualitative research Grounded theory research
			Purposive sample
			19 providers (12 RNs, 2 CNMs, and 5 MDs.) Observation of 10 of the 19 participants (7 RNs, 2 MDs, and 1 CNM).
Mallidou et al., [30] Canada	Nurses in 12 hospitals	Relationships and mechanisms between nursing specialty subcultures and selected patient outcomes	Non-randomised cross sectional and secondary data analysis
			Sample 1937 nurses
McKenna, Smith et al. [31] New Zealand	Nurses registered to practice in the previous year	Horizontal violence experiences of newly registered nurses	Non-randomised cross sectional survey with open ended responses
			Sample 584 Registered Nurses
MacKusick and Minick [32] United States	Registered nurses	Identify the factors influencing the decision of RNs to leave clinical nursing practice	Phenomenological design
			Purposive sample n = 10
McCusker et al., [33] Canada	Nurses in 13 units in one hospital	Confirm sub-scales from (NWI-R) assess the nursing work environment	Non-randomised cross sectional
			Sample 283
Randle [34] United Kingdom	Student nurses in one program	Influence of pre-registration experiences on self-esteem	Qualitative research - Grounded theory
			56 students at commencement, 39 at conclusion
Rice Simpson and Lyndon, [35] United States	Midwives obstetric nurses one metropolitan area	Describe how nurses would respond in common clinical situations	Non-randomised cross-sectional survey with open ended response
			Sample 704
Roche 2010 [44] Australia	94 nursing wards in 21 hospitals between 2004 and 2006	Relationships between nurses’ self-rated perceptions of violence, and the nursing working environment and patient outcomes	Non-randomised cross-sectional survey and secondary analysis of data
			Sample 3,099
Rosenstein, [15] United States	Health workers 142 acute hospitals	Relationships between nurse-physician relationships and nurse satisfaction and retention	Non-randomised cross-sectional survey
			Sample 2562
			Nurses = 1615
			Physicians = 389
			Executives = 104
Rosenstein and O’Daniel [8] United States	Large multi facility health care network	Investigate prevalence and impact of disruptive behaviour on clinical outcomes	Non-randomised cross-sectional survey with open ended responses
			Sample 1509 (1091 RN, 402 physicians, 16 administrators survey first distributed in 2003 and ongoing
Rosenstein and O’Daniel [36] United States	Clinical staff in Four VHA regions	Significance of disruptive behaviour on communication and collaboration and impact on patient care	Non-randomised cross-sectional survey with open ended responses
			Sample 4,500 participants completed survey (2,846 nurses, 944 physicians, 40 executives, 700 not specified)
Rosenstein and O’Daniel [36] United States	Staff in large metropolitan academic medical centre	Disruptive behaviours in peri operative services	Non-randomised cross-sectional survey with open ended responses
			Sample 244 professional staff (82 MDs, 71 RNs, 24 nurse anesthetists, 18 surgical technologists, and 49 others)
Sofield and Salmond [37] United States	Nurse in three-hospital health system	Nurses experiences of verbal abuse and association with intent to leave	Randomised cross sectional survey
			Sample 465
Simons and Mawn [38] United States	Newly registered nurses in one state	Investigation of bullying among newly registered nurses	Non-randomised cross sectional survey with open ended response
			Sample 511
Strauss [39] United States	CRNAs in one state	Investigated nurses exposure to 20 types of bullying behaviour by physicians	Randomized cross-sectional survey with open ended responses
			Sample size not specified
Smith [40] United States	Peri-operative nurses	Relationship between bullying and patient outcomes in terms of five surgical never events	Non-randomised cross sectional survey
			Sample 853
Walrath, Dang et al. [41] United States	Registered nurses 1 hospital	Nurses experiences of disruptive clinician behaviour	Qualitative study
			Purposive sample of 96 RNs
Weisbrod [42] United States	Students nurses one university program	Perceptions of violence in the clinical setting	Mixed method
			Non-randomised cross sectional survey with open ended responses & focus groups. Sample 37

### Search outcome

The studies were undertaken in a variety of healthcare settings in a number of countries and the nature of professional behaviours and their association with patient care was either explicitly or indirectly investigated. Country of origin of the studies were primarily the United States (n = 16), Australia (n = 7) and Canada (n = 3). The majority of the studies involved investigating the experiences of nurses employed in hospitals or large health centres (n = 16) or nurses in specific states or regions (n = 3), while two studies investigated students enrolled in nursing programs. Eighteen studies focused upon investigating various forms of hostile clinician behaviours categorised as disruptive behaviours, intimidation, horizontal violence and bullying, aggression, and verbal abuse. Six studies examined features of the nursing workplace, including nurse-physician collaboration and teamwork and associations with standards of care or specific adverse events. The remaining studies examined issues such as intention to leave, ethical and legal frameworks informing practice and factors that influence nurses self-esteem, agency and clinical reasoning.

### Quality review

A meta-analysis of the quantitative data was not feasible due to the heterogeneity of behaviours and outcome measures investigated. The JBI-MAStari appraisal instrument for descriptive and case study designs guided the appraisal of quantitative studies. During this appraisal studies were excluded from the review on the basis of poor methodological quality if they scored less than 4 using the MAStari checklist. Based on assessed points, each reviewed study fell into one of three categories: low (score = 4–5), moderate (scores = 6–7) or high (scores = 8 or >). To assess the quality of studies reporting qualitative data the ten item JBI-QARI checklist from the Joanna Briggs Institute [45] was employed. Studies were excluded from the review on the basis of poor methodological quality if they scored less than 4 using the QARI checklist. The criteria on the tool relate to establishing the nature and appropriateness of the qualitative methodology. Based on assessed points, each reviewed study fell into one of three categories: low (score = 4–5), moderate (scores = 6–7) or high (scores = 8 or >). As no standard valid appraisal tool for mixed methods reviews was identified at the time of the review, templates from the standardised critical appraisal instruments from the Joanna Briggs Institute were employed [45]. Table 2 presents the appraisal instrument criteria, and the quality review findings for each criterion according to the category of study. Overall, for the qualitative studies 4 were assessed to be of a high quality, 3 were moderate and 3 low. Whilst 10 of the quantitative studies were assessed to be of low quality, 6 of moderate quality and 3 of high quality, for the mixed methods studies 1 was deemed to be low quality and 1 high quality.

**Table 2:** Quality review criteria and summary of findings

**Criteria**	**No of studies**			
	**Yes**	**No**	**Unclear**	**N/A**
**Quantitative studies**				
*Design and sample*				
Random or probability sample	1	17		
Sample adequate size and representative	17		1	
Inclusion criteria clearly defined	4	12	2	
*Measurement*				
Valid and reliable measures	4	12	2	
Confounding factors identified and managed	4	14		
*Statistical analysis*				
Appropriate statistics	18			
**Qualitative studies**				
*Study design*				
Congruity between philosophical perspective & methodology	6		4	
Congruity between research methodology and research question	6		4	
Congruity between the research methodology & data collection methods	7		2	1
Participants and their voices are adequately represented	5	5		
Influence of the researcher is addressed	5	5		
Statement locating the researcher culturally or theoretically	4	6		
*Analysis*				
Congruity between the research methodology & interpretation of results	5	2	2	
Congruity between research methodology & presentation/ analysis	6	1	3	
*Presentation of findings*				
Accords with current ethical criteria , evidence of ethical approval	7		3	
Conclusions drawn flow from the data	6	2	2	
**Mixed Methods**				
*Study design*				
Random or probability sample		2		
Sample adequate size and representative	1	1		
Confounding factors identified and managed		2		
Mixed methods design is relevant to address the research question	2			
*Measurement*				
Valid and reliable measures		2		
*Analysis*				
Influence of the researcher is addressed		2		
Conclusions drawn flow from the data		1	1	
Appropriate consideration given to the limitations of the method		1	1	

### Summary of quality review

All studies involving human subjects had obtained institutional ethics approval. The most common limitation of the quantitative studies related to sampling and study design. The majority employed non-random samples and are rated at risk of selection bias and limited inference on causality. The limited use of randomisation in dealing with confounding factors reduces the generalisability of findings. Of the two studies that employed randomisation, this was not reported in sufficient detail to be rated as adequate to establish control of selection bias. A further limitation of the majority of the correlational studies was the use of nurses’ self-reported perceptions on quality of care and exposure to hostile behaviours, with the recall period varying from the preceding year or for an undefined period. Distinguishing features between the high and medium rated quantiative studies was the detail on inclusion criteria for the sample and the reliability of measuring effects in the higher rated studies. Quantitative studies investigating features of the nursing work environment employed measures with established construct validity; in contrast few of the studies specifically examining hostile behaviours employed validated measures of these consturcts. Of note, six of the quantitative studies assessed to be of low quality reported little or no detail on the survey method. Descriptive statistics, correlation and regression analysis were the more common statistical analysis. Response rates in the quantitative studies varied from 37.0% to 80.3%. Of the survey studies reporting qualitative data, one was rated moderate quality; the majority of these studies provide little detail on the process for refining categories and themes for the open-ended responses. The absence of this information made it impossible to evaluate the presence or extent of reporting bias in the qualitative data presented from the majority of these studies.

The more common limitation of qualitative studies was related to the congruence between the study methodology and representation and interpretation of data. Although the majority of qualitative studies reported several methods to increase trustworthiness of the analysis, including full transcription of audio taped interviews and member checking, little detail was provided in a number of studies. Three qualitative studies were rated low. These studies demonstrated little congruence between the methodology and analysis and interpretation of data. The authors also provided little detail on analysis methodology and it was not possible to determine concepts such as trustworthiness of the analysis.

### Analysis

Using content analysis [46] the care from the studies were aggregated into categories based on common concepts and experiences that revealed the detail of hostile clinician behaviours and its association with patient care. Initially, each manuscript was read several times and findings that identified relationships between hostile clinician behaviours and care were extracted and compiled in a tabular format. This resulted in a total of 11 findings from the quantitative studies and 144 sections of narrative data from the qualitative studies. These extracted findings were sorted into thematic categories based on common characteristics to arrive at a synthesis [47]. This process allowed for the identification of the nature of relationship between hostile clinician behaviours and patient care [48]. To ensure the accuracy of the analysis cross member checking was undertaken and coding revisions were made by the two reviewers to ensure a shared understanding and provide confirmation of the analysis [49].

## Results

Through aggregating the data in this way, four themes emerged: (1) Physician-nurse relations and patient care, (2) Nurse-nurse bullying, intimidation and patient care, (3) Reduced nurse performance related to exposure to hostile clinician behaviours, and (4) Nurses and physicians directly implicating patients in hostile clinician behaviours. The details of each of these themes are presented below.

### Physician-nurse relations and patient care

Physician behaviours and physician-nurse relations were specifically examined in fourteen studies in this review [8,15,22-25,30,33,35,36,39-41]. Of this group of studies, one was qualitative [41] and the remainder were cross sectional designs, of which four employed secondary patient care outcome data collected from hospitals surveys or incident report systems [22,24,25,30] and nine employed nurse self-reported perceptions of quality of care, perceptions of the impact of hostile behaviours upon nursing care and adverse events [8,15,23,33,35,36,39,40]; Unfavourable incidents investigated in these studies included: adverse events [36], patient mortality [36,40], surgery on the wrong site or wrong patient (Smith [40]), nurse non-adherence to evidence standards and protocols [35], medication errors [22], and compromise in patient care or safety [36,37].

Particular care of interest in these studies was the degree to which satisfaction with nurse-physician relations were associated with nurse reported quality of care and the occurrence of adverse events. McCusker et al. [33] reported that nurse self-reports of verbal abuse and reduced satisfaction with nurse-physician relations was associated with a lower overall quality of nursing care. Similarly, Mallidou et al*.*, [30] reported that good relationships between nurses and physicians was associated with improved quality of care and reduced adverse patient events.

Other studies reported that physician intimidation played a role in medication errors and contributed to nurses’ failure to clarify medication orders for which they held concerns [8,27,36]. Also of note were the findings of Smith [40] who identified statistically significant relations between exposure to bullying and an increase in the number of adverse patient care in the peri-operative setting including surgery on the wrong patient (R = .25, p < 0.001.) and retained surgical items (R = .26, p < 0.001). It was not possible to draw firm conclusion for one of the manuscripts [39] as there was little explanation of statistical analysis undertaken to support the finding that abusive physician behaviour resulted in increased errors.

Nine of the studies examining nurse-physician relations and patient care employed either the Practice Environment Scale of the Nursing Work Index (PES-NWI) [22,24], the revised Nursing Work Index (NWI-R) [23,30,33]), or the Impact of Disruptive Behaviour on Patient Care tool [8,15,36]. These studies had mixed results; with four studies reporting nurse-physician relations were statistically significant in their negative relation to quality of patient care [23,25,30,33], two reporting that physician-nurse relations were not associated with adverse events [24,33], and one study reported reporting a weak positive association between a positive practice environment and medication errors (R0.15, *P <* .01) [22]. In examining the association between nursing work environment (including collegial nurse-physician relations and team member interactions) and medication errors per 1000 patients, the study by Fasolino and Snyder [22] reported summed scores on the PES-NWI, hence it is not possible from this study to infer specific detail on the nature of the relation between medication error and nurse-physician relationships. In the study undertaken by Gunnarsdo’ttir and colleagues [23] nurse self-reported quality of care was independently associated with nurse–doctor relations, with a one-point increase in nurse–doctor relations associated with nearly a doubling of the odds of nurse’s reporting excellent quality of care. In contrast, employing data from an annual hospital survey Hanrahan et al., [24] reported that nurse-physician relations were not significant in their relationship with adverse events. Instead, the findings from this study identified that nurse physician relationships were highly significant and poor working relations were associated with work-related injuries. Of note, the majority of these studies relied on nurse self-report, with three studies employing data collected through incident report or hospital care survey data [22,24,30].

Four qualitative studies [26,29,41,43] and six survey studies reporting qualitative open end response data [8,36] reported on the relation between nurse-physician relationships and care. In these studies, physicians were reported as refusing to listen to requests or information regarding changes in the condition of patients from nursing staff [41], with refusal accompanied by hostile behaviour such as verbal abuse, sarcasm, rude, demeaning, belittling or dismissive comments, and intimidation such as swearing, using a raised voice or throwing items [8,15,36,39,43] (that interrupted concentration [26] or reduced nurses capacity or willingness to take a stand on issues of concern [50]. In some instances the hostility was reported to be repeated while the patient’s condition deteriorated [8,15,36]. In other instances intimidation from physicians involved repeatedly ignoring calls or failing to act on calls/requests without overt aggression being directed towards the nurse that placed patients at risk [8,36].

### Nurse-nurse bullying, intimidation and patient care

Nurse-nurse bullying and intimidation and its relation with care was specifically reported in ten studies [19,20,28,31,32,36,38,39],[41,42]. Five of these studies involved cross-sectional surveys of bullying or aggression with open response options that provided nurse self-report perceptions of the way in which nurse-nurse bullying and intimidation impacted patient care [19,31,36,38,39,42]. Other studies in this group were qualitative and collected nurses experiences of whistlebloweing, bullying and aggression through in-depth interviews [20,28,32]. These studies reported nurse-nurse hostile clinician behaviours extended to creating risks for patients by implicating clinical care in acts of sabotage or payback between nurses [20,28,32]. The types of behaviours cited as a feature of this form of hostile clinician behaviour included withholding or refusing to pass on relevant clinical information with the intention of making work difficult [28] or placing an individual nurse under pressure or forcing clinical errors [4,20,32]. At times these acts occurred as a form of payback for transgressing an “accepted” workgroup norm, or were directed towards nurses who had spoken out about concerns they held about care quality or practices [4,20,32].

In three survey studies [19,31,38] and two qualitative studies [20,32] nurse-to-nurse hostility was reported to result in individuals feeling overwhelmed, unable to ask for help, feeling out of their depth with patient situations, fearful of making errors or causing harm, and unable to trust. The tendency to ignore requests for assistance from colleagues, especially in situations where assistance was needed to ensure patient safety or to respond to complex or demanding clinical situations was reported by respondents in four survey studies [19,31,38,42] and three qualitative studies [20,32,41].

### Reduced nurse performance related to exposure hostile clinician behaviours

An additional focus in a number of studies reviewed was the extent to which hostile clinician behaviours impacted features of staff performance with the potential to influence patient care. In the survey study by Strauss [39] respondents recounted that in situations characterised by repeated physician initiated hostile clinician behaviours and failure to respond they persisted in repeatedly engaging with hostile physicians in an attempt to amend treatment orders or ensure appropriate treatment interventions. In contrast, insights from three qualitative studies detailed that nurses who had experienced or witnessed hostility from physicians engaged in avoidance and delayed communication [26,29] or were less likely to take a stand on clinical issues of concern [29]. Another qualitative study drew attention to the way in which nurse-to-nurse hostile behaviours eroded teamwork, morale, and trust among nurses [28].

In addition, in five survey studies that included analysis of narrative responses, nurse avoidance and delayed communication was described to place patients at increased risk or delay treatment by impacting negatively upon the flow of clinical information and patient care [8,36-38]. In two of these studies, nurses who experienced hostile clinician behaviours noted it had the effect of distracting them from their work and reducing their ability to concentrate [8,37] these interruptions to concentration were a cause of minor patient care errors. Hostile clinician behaviours were reported to result in avoidant behaviours from nurses in four survey studies in this group [8,36,37]. In these four survey studies, nurse avoidance was reported to extend to a complete failure to communicate, in some instances over extended periods in situations when nurses were intimidated and afraid to call physicians who had a reputation of hostility, or from whom they had witnessed hostility in the past. In the study by Sofield & Salmond [37] nurse self-doubt stemming from hostile behaviours led to avoidance which was characterised by despondency and complacency, with nurses unable to problem solve or follow through with care resulting in errors or near misses. The studies by Rosenstein [36] and Sofield & Salmon [37] reported nurse-to-nurse hostile behaviours impacted negatively upon teamwork, morale, and trust among colleagues.

The study by the Institute of Safe Medication Practices [27] was one of the four survey studies to attempt to determine mechanisms for the way in which hostile behaviours impacted patient care [27,35,37,51]. In the Institue of Safe Medication Practices [27] survey the authors included questions regarding nurses’ experiences of avoiding seeking clarification for medication orders for which they held concerns. Noteworthy in this study intimidation was reported to result in nurses feeling pressured to accept an order even though they felt the order was incorrect. In another study in this group, intimidation resulted in nurses going against what they knew to be current evidence and best practice [35]. The remaining two studies in this group examined the association between nurses’ experience of verbal abuse [51] and reduced morale, intent to leave, productivity [37], and caregiving error [51]. Results indicated that in one study 67% of respondents’ perceived verbal abuse impacted upon morale [37] while in the other study 13% of nurses reported making caregiving errors they attributed to the abuse [21]. Of note, in this study although the more common source of verbal abuse was from other nurses and physicians, the authors do not report the source of abuse that contributed to error. Additionally, in the study by Sofield & Salmon [37] 41% reported verbal abuse impacted upon productivity, while abuse and intent to leave were significantly related (*r* = 211, *p* ≤ 0.01). The study did not examine the nature of these relations in any detail.

### Nurses and Physicians directly implicating patients in hostile clinician behaviours

Five studies reported the ways in which patients were directly implicated in hostile clinician behaviours [8,34,36,39]. In other studies the potential for hostile behaviour to disrupt care was identified [41]. Both nurses and physicians were reported to engage in verbal abuse of other nurses in front of patients, this was perceived by nurses to erode patient confidence in the capability of the nurse targeted [8,36,39]. In other instances, nurses described that hostility from physicians towards nursing could implicate patients through hostility that was initially directed at the nurse being turned upon the patient [8,39]. In other situations it was reported nurses abused their power over patients through yelling, swearing or withholding privileges from vulnerable patients [21,34].

## Discussion

The findings of this review lend support to the claims that hostile clinician behaviours can impact unfavourably upon patient care. Importantly, understanding the nature of this relationship in any detail is made difficult due to the variability in study designs and the small number of studies identified to be of high quality. Overall, we found little robust evidence detailing the nature and extent of the relationship between hostile clinician behaviours and patient care, the evidence is mostly small in size and weak to moderate in quality. It is perhaps important to note that studies rated as low in this review [8,15,19,27,36,38,39,51] are commonly employed to substantiate the impact of hostile clinician behaviours on patient care [14,52].

It is important to note that while a small group of highly rated survey studies employed valid and reliable instruments, and a few included reliable secondary data sources to identify the association between nurse-physician relations and patient care [22,24,30], none of these studies specifically examined hostile behaviours. While findings from these studies indicate that nurse satisfaction with nurse-physician relationships is associated with the quality of patient care, the presence or absence of hostility can only be inferred in these studies from items measuring global perceptions of physician-nurse working relationships. While proxy measures of quality of care suggest that patients fared worse in environments where nurse-physician relationships were rated lower or nurses’ experienced verbal abuse, the reliability of nurse recall of adverse care outcomes and their association with hostile clinician behaviours may be inaccurate.

The findings from our review indicate that both nurse-nurse and nurse-physician hostile behaviours have the potential to impact on patient care and care. The types of hostile behaviours reported ranged from isolating a colleague and refusing them assistance through to directly involving patients in the abuse. Importantly, while hostile clinician behaviours experienced by nurses are widely recognised to include behaviours such as overt aggression and intimidation, this review has drawn attention to the place of more subtle behaviours and their potential to impact on patient care. An important contradiction was evidenced through the review, the majority of survey studies focused upon examining nurse-physician hostile relationships at the exclusion of nurse-nurse relationships. We found no rigorously designed and executed studies that examined nurse initiated hostile behaviours and their relationship with patient care. This is remarkable, particularly given the extensive evidence regarding the prevalence and nature of nurse-nurse hostile behaviours.

### Implications for further research

Although there is evidence that nurse-assessed quality of care measures when validated against independent measures of patient mortality have been found to be a good predictor of actual nursing outcomes [53], confirmation of the association between care outcomes and hostile behaviours requires larger more rigorously designed studies that specifically identify the nature and extent of the relationship. In further examining the impact of hostile behaviours and the nursing work environment or clinical outcomes it is important that nurse researchers direct attention towards addressing the limitations of the predominant focus upon nurse-physican relationships at the exclusion of consideration of nurse-nurse relationships. Furthermore, the use of tools designed to measure global nurse-physician relationships [22,23,30,33] or the use of non-validated instruments measuring nurse and physician behaviours [8,15,36] suggests that there is little consensus or theoretical development to inform conceptualisation of professional relationships and the nursing work environment. While the NWI and its derivates are widely employed instruments in nursing research [53], it has not been established whether the global measure of physician-nurse collegiality and teamwork in the NWI and its derivates are an appropriate measure of these constructs [54].

Of note, we failed to find survey studies that employed instrumentation sufficiently refined to capture the breadth of hostile behaviours between clinicians reported in the qualitative studies reviewed. This is a notable observation and may reflect the country of origin of the studies; the majority of qualitative studies were undertaken in Europe and Australia while instrumentation and survey studies largely emanated from North America. Drawing upon our findings, future research may modify existing instruments or develop new measures to more specifically explore the relations between hostile clinician behaviours and clinical outcomes. To strengthen study designs, future research can utilise more probability sampling and analysis of secondary data sources for clinical outcomes. The application of higher level multivariate statistical analysis, such as structural equation modelling can be utilised to understand the relationships between hostile behaviours, other workplace factors and clinical outcomes. These studies might usefully shed light on potential intervention strategies.

### Limitations

An important limitation identified through this review is that no studies have specifically examined hostile behaviours and reliable secondary sources of outcome data. Furthermore, the variability in the conceptualisations of hostile clinician behaviours and the associated measurement instruments may limit the validity of the findings.

## Conclusion

It is evident from this review that the implications of hostile clinician behaviours upon patient care and patient care processes are potentially profound and more complex than often postulated. The paucity of robustly designed studies indicates that this is a comparatively under researched area and findings from this review suggest this may be a significant omission in our understanding of the factors that influence nurses work environment and patient care. The findings from the review provide the basis for further investigation or the development of conceptual models and working hypothesis to extend understanding of this phenomenon and guide the further development of intervention strategies.

## Competing interests

The authors declare that they have no competing interests.

## Authors’ contributions

MH and JD participated in design of the study, MH undertook the literature search, MH and DJ participated in manuscript reviews, MH and JD drafted manuscript. Both authors read and approved the final manuscript.

## Pre-publication history

The pre-publication history for this paper can be accessed here:

http://www.biomedcentral.com/1472-6955/12/25/prepub

## References

[B1] Mikkelsen EG Einarsen S Basic assumptions and symptoms of posttraumatic stress among victims of bullying at work Eur J Work Organ Psych 2002 11 1 87 111 10.1080/13594320143000861

[B2] Katrinli A Atabay G Gunay G Cangarli BG Nurses’ perceptions of individual and organisational political reasons for horizontal peer bullying Nurs Ethics 2010 17 5 614 627 10.1177/0969733010368748 20801963

[B3] Robertson N Perry A Institutionally based health care workers’ exposure to traumatogenic events: systematic review of PTSD presentation J Traum Stress 2010 23 3 417 420 10.1002/jts.20537 20564377

[B4] Jackson D Clare J Mannix J Who would want to be a nurse? Violence in the workplace – a factor in recruitment and retention J Nurs Manage 2002 10 1 13 20 10.1046/j.0966-0429.2001.00262.x 11906596

[B5] Mikkelsen EG Einarsen S Relationships between exposure to bullying at work and psychological and psychosomatic health complaints: the role of state negative affectivity and generalised self-efficacy Scand J Psych 2002 43 5 397 405 10.1111/1467-9450.00307 12500778

[B6] Royal College of Nursing Working well: a call to employers 2002 London, UK: Royal College of Nursing

[B7] Hutchinson M Vickers M Wilkes L Jackson D A typology of bullying behaviours: the experiences of Australian nurses J Clin Nurs 2010 19 2319 2328 10.1111/j.1365-2702.2009.03160.x 20659206

[B8] Rosenstein AH O’Daniel M Disruptive behaviour and clinical care: perceptions of nurses and physicians Am J Nurs 2005 105 1 54 64 10.1097/00000446-200501000-00025 15659998

[B9] Celik SS Celik Y Ağirbaş I Uğurluoğlu O Verbal and physical abuse against nurses in Turkey Int Nurs Rev 2007 54 4 359 366 10.1111/j.1466-7657.2007.00548.x 17958665

[B10] Woelfle CY McCaffrey R Nurse on nurse Nurs Forum 2007 42 3 123 131 10.1111/j.1744-6198.2007.00076.x 17661804

[B11] Kidd JD Finlayson MP Mental illness in the nursing workplace: a collective auto ethnography Contemp Nurse 2010 36 1 2 10.5172/conu.2010.36.1-2.021 21254820

[B12] Vessey JA DeMarco R DiFazio R Bullying, harassment, and horizontal violence in the nursing workforce: the state of the science Annu Rev Nurs Res 2011 28 133 157 21639026 10.1891/0739-6686.28.133

[B13] Longo J Sherman R Levelling horizontal violence Nurs Manage 2007 38 3 34 37 10.1097/01.NUMA.0000262925.77680.e0 17491129

[B14] The Joint Commission Behaviors that undermine a culture of safetyvol. Sentinel Event Alert #40 2008 Available at: http://www.jointcommission.org/SentinelEvents/SentinelEventAlert/sea_40.htm 18686330

[B15] Rosenstein A Nurse--physician relationships: impact on nurse satisfaction and retention Am J Nurs 2002 102 6 26 34 10.1097/00000446-200206000-00040 12394075

[B16] The Joint commission Leadership standard clarified to address behaviors that undermine a safety culture Joint Comm Persp 2011 32 1 http://www.jointcommission.org/assets/1/6/Leadership_standard_behaviors.pdf accessed 10/10/12 22360128

[B17] Saxtone R Hines T Enriquez M The negative impact of nurse-physician dirsruptive behaviour on patient safety: a review of the literature J Patient Saf 2009 5 3 180 183 10.1097/PTS.0b013e3181b4c5d7 19927052

[B18] Pace R Pluye P Bartlett G Macaulay AC Salsberg J Jagosh J Seller R Testing the reliability and efficiency of the pilot mixed methods appraisal tool (MMAT) for systematic mixed studies review Int J Nurs Stud 2012 49 47 53 10.1016/j.ijnurstu.2011.07.002 21835406

[B19] Curtis J Bowen I Reid A You have no credibility: nursing student’s experiences of horizontal violence Nurs Edu Pract 2007 7 156 163 10.1016/j.nepr.2006.06.002 17689439

[B20] Corney B Aggression in the workplace: a study of horizontal violence utilising Heideggerian hermeneutic phenomenolog J Health Organ Manage 2008 22 2 164 177 10.1108/14777260810876321 18700526

[B21] Farrell GA Aggression in the clinical setting: Nurses’ views J Adv Nurs 1997 25 501 508 10.1046/j.1365-2648.1997.1997025501.x 9080276

[B22] Fasolino T Snyder R Linking nurse characteristics, team member effectiveness, practice environment, and medication error incidence J Nurs Care Qual 2012 27 2 1 8 10.1097/NCQ.0b013e31823e827a 22218262

[B23] Gunnarsdo’ttir S Clarke SP Rafferty AM Nutbeam D Front-line management, staffing and nurse–doctor relationships as predictors of nurse and patient care. A survey of Icelandic hospital nurses In J Nurs Stud 2009 46 920 927 10.1016/j.ijnurstu.2006.11.007 17229425

[B24] Hanrahan NP Kumar A Aiken LH Adverse events associated with organizational factors of general hospital inpatient psychiatric care environments Psychiatr Serv 2010 61 6 569 574 10.1176/appi.ps.61.6.569 20513679 PMC2890256

[B25] Tervo-Heikkinen T Partanen P Aalto P Vehvilainen-Julkunen K Nurses’ Work environment and nursing care: a survey study among Finnish university hospital registered nurses Int J Nurs Pract 2008 14 365 375 10.1111/j.1440-172X.2008.00707.x 18808536

[B26] Higgins BL MacIntosh J Operating room nurses’ perceptions of the effects of phsyician-perpetrated abuse Int Nurs Rev 2010 57 321 327 10.1111/j.1466-7657.2009.00767.x 20796061

[B27] Institute for Safe Medication Practices Intimidation: practitioners speak up about this unresolved problem (part I) Safety Alert 2004 http://www.ismp.org/Newsletters/acutecare/articles/20040311_2.asp

[B28] Jackson D Peters K Andrew S Edenborough M Halcomb E Luck L Salamonson Y Weaver R Wilkes L Trial and retribution: a qualitative study of whistle blowing and workplace relationships in nursing Contemp Nurs 2010 36 1 34 44 10.5172/conu.2010.36.1-2.034 21254821

[B29] Lyndon A Social and environmental conditions creating fluctuating agency for safety in two urban academic birth centers J Obstet Gynecol Neonatal Nurs 2008 37 1 13 23 10.1111/j.1552-6909.2007.00204.x 18226153

[B30] Mallidou AA Cummings GC Estabrooks CA Giovannetti PB Nurse specialty subcultures and patient care in acute care hospitals: a multiple-group structural equation modeling Int J Nurs Stud 2011 48 81 93 10.1016/j.ijnurstu.2010.06.002 20598308

[B31] McKenna B Smith NA Poole SJ Coverdale JH Horizontal violence: experiences of registered nurses in their first year of practice J Adv Nurs 2003 42 1 90 96 10.1046/j.1365-2648.2003.02583.x 12641816

[B32] MacKusick CI Minick P Why are nurses leaving? Findings from an initial qualitative study on nursing attrition Med Surg Nurs 2010 19 6 335 339 21337990

[B33] McCusker J Dendukuri N Cardinal L Laplante J Bambonye L Nursing work environment and quality of care: differences between units at the same hospital Int J Health Care Qual Assur 2004 17 6 313 322 10.1108/09526860410557561 15552386

[B34] Randle J Bullying in the nursing profession J Adv Nurs 2003 43 4 395 401 10.1046/j.1365-2648.2003.02728.x 12887358

[B35] Rice Simpson K Lyndon A Clinical disagreements during labor and birth: How does real life compare to best practice? MCN Am J Matern Child Nurs 2009 34 1 31 39 10.1097/01.NMC.0000343863.72237.2b 19104317

[B36] Rosenstein AH O’Daniel M Impact and implications of disruptive behavior in the perioperative arena J Am College Surg 2006 2006 3 27 10.1016/j.jamcollsurg.2006.03.027 16798492

[B37] Sofield L Salmon SW Workplace violence: a focus on verbal abuse and intent to leave the organisation Orthop Nurs 2003 22 4 274 283 10.1097/00006416-200307000-00008 12961971

[B38] Simons S Mawn B Bullying in the workplace - a qualititative study of newly licenced registered nurses Am Assoc Occup Health Nurs 2010 58 305 311 10.3928/08910162-20100616-02 20608570

[B39] Strauss S A study on physician buliying as gender harassment to female and male operating room nurses in Minnesota (Part I) Minn Nurs Accent 2008 Sept/Oct 14 16

[B40] Smith J Bullying in the nursing workplace: a study of perioperative nurses 2012 Charleston, United States: BiblioLabsII

[B41] Walrath JM Dang D Nyberg D Hospital RNs’ experiences with disruptive behavior a qualitative study J Nurs Care Qual 2010 25 105 116 10.1097/NCQ.0b013e3181c7b58e 19935429

[B42] Weisbrod M Nursing students’ perceptions of workplace violence: a feminist research study 2007 Athabasca AB: Athabasca University

[B43] Chairella M McInnes E Legality, morality and reality - the role of the nurse in maintaining standards of care Aust J Adv Nurs 2008 26 1 77 83

[B44] Roche M Violence toward nurses, the work environment, and patient outcomes J Nurs Scholarsh 2010 42 1 13 18 10.1111/j.1547-5069.2009.01321.x 20487182

[B45] The Joanna Briggs Institute Joanna Briggs Institute Reviewers’ Manual 2011 Adelaide, Australia: The Joanna Briggs Institute, the University of Adelaide

[B46] Krippendorff K Content analysis: an introduction to its methodology 2004 2 Thousand Oaks, California: Sage Publications

[B47] Pearson A Balancing the evidence: incorporating the synthesis of qualitative data into systematic reviews JBI Reports. vol. 1 2004 Adelaide, Australia: Joanna Briggs Institute 45 64

[B48] Thomas J Harden A Methods for the thematic synthesis of qualitative research in systematic reviews BMC Med Res Methodol 2008 8 45 10.1186/1471-2288-8-45 18616818 PMC2478656

[B49] Coffey A Atkinson P Making sense of qualitative data: complimentary research strategies 1996 Thousand Oaks, California: Sage Publications

[B50] Lyndon A Perinatal safety: from concept to nursing practice J Perinat Neonat Nurs 2010 24 1 22 31 10.1097/JPN.0b013e3181cb9351 PMC2921888 20147827

[B51] Rowe MM Sherlock H Stress and verbal abuse in nursing: Do burn out nurses eat their young? J Nurs Manage 2005 13 242 248 10.1111/j.1365-2834.2004.00533.x 15819837

[B52] Vessey JA Demarco R DiFazio R Bullying, harassment, and horizontal violence in the nursing workforce: the state of the science Ann Rev Nurs Res 2010 28 133 157 10.1891/0739-6686.28.133 21639026

[B53] Aiken LH Clarke SP Sloane DM Hospital staffing, organization, and quality of care: cross-national findings Nurs Outlook 2002 50 187 194 10.1067/mno.2002.126696 12386653

[B54] Bonneterre V Liaudy S Chatellier G Lang T de Gaudemaris R Reliability, validity, and health issues arising from questionnaires used to measure psychosocial and organizational work factors (POWFs) among hospital nurses: a critical review J Nurs Measure 2008 16 204 230 10.1891/1061-3749.16.3.207 19886473

